# A Paired Flow Cytometry–Pathology Assessment for Immune Cell Detection in Intestinal Biopsies: Proof of Principle

**DOI:** 10.3390/mps8050122

**Published:** 2025-10-16

**Authors:** Alexandros Skamnelos, Georgios S. Markopoulos, Lefkothea Dova, Ioulia Tragani, Meropi Katsipaneli, Dimitrios Christodoulou, Konstantinos Katsanos, Evangeli Lampri

**Affiliations:** 1 Department of Gastroenterology, University Hospital of Ioannina, 45110 Ioannina, Greece; alskamnelos@gmail.com (A.S.); dchristo@uoi.gr (D.C.); kkatsanos@uoi.gr (K.K.); 2 Unit of Molecular Biology and Translational Flow Cytometry, University Hospital of Ioannina, 45110 Ioannina, Greece; thea_dova@yahoo.gr; 3 Laboratory of Physiology, Faculty of Medicine, University of Ioannina, 45110 Ioannina, Greece; 4 Department of Pathology, University Hospital of Ioannina, 45110 Ioannina, Greeceirokts7@gmail.com (M.K.)

**Keywords:** flow cytometry, immunohistochemistry, colonic biopsies, immune profiling, IBD, lymphocytes, tissue digestion

## Abstract

Accurate quantification of immune cell subpopulations is essential for understanding immune responses in research and clinical settings. Flow cytometry (FC) is widely used for immune cell phenotyping, providing rapid and quantitative single-cell resolution. However, tissue-based pathological assessment offers additional spatial and morphological context that is often necessary for a comprehensive understanding of immune cell distribution. Traditionally, these methods are applied separately to different specimens, limiting direct comparative analysis. Here, we describe a simple combined approach to immune cell quantification that integrates both FC and pathology analysis within the same tissue specimen of colon biopsies. Tissue samples were divided into two portions: one processed into a single-cell suspension for FC-based characterization of CD45^+^, CD3^+^, CD4^+^, and CD8^+^ T cells and another formalin-fixed, paraffin-embedded (FFPE), and analyzed with hematoxylin and eosin (H&E) for eosinophils and immunohistochemistry (IHC) for CD4 and CD8. A pilot analysis of 10 samples shows high concordance of the results taken from the two methods, allowing for cross-validation between methodologies and improved diagnostic accuracy. This proof-of-principle study demonstrates the feasibility of an integrated workflow that combines FC and pathology for immune cell quantification, which provides assessment of immune cell populations from the limited material of intestinal biopsies with potential for improved diagnostic accuracy.

## 1. Introduction

Accurate immune cell population quantification is crucial for diagnosis and understanding the biology underlying immune responses in infections, inflammation, and cancer, among others. Notably, immune populations exhibit a tissue-specific pattern [1]. Flow cytometry (FC), a technology of choice for the quantification of cellular phenotype [2], enables rapid and high-throughput analysis of immune cell subpopulations using antibodies conjugated with fluorochromes [3]. While FC provides excellent single-cell resolution and quantitative data, it lacks spatial context, which is crucial for understanding tissue microenvironments, a feature only available in imaging flow cytometry [4].

Conversely, pathology with histological and immunohistochemical techniques gives information about the localization of immune cells within tissue architecture, but quantitative analysis is not automated. Traditional approaches analyze immune populations via either flow cytometry or histological staining alone.

By integrating FC with histopathology, we aim to establish a paired, correlative immune-profiling approach, i.e., splitting the same biopsy to quantify identical T-cell markers by both modalities and statistically compare (correlate) the resulting metrics. Understanding the immune cell composition in colonic mucosa is crucial for elucidating mechanisms of pathology in various diseases. For example, in inflammatory bowel disease (IBD), studies have shown that the CD4:CD8 ratio in intestinal tissues may play a significant role in the pathogenesis of the disease. For instance, research indicates that patients with IBD exhibit increased activation and proliferation of both CD4+ and CD8+ T cells in the intestinal mucosa, leading to an imbalance in the CD4:CD8 ratio. This altered ratio is associated with disease activity and may contribute to the chronic inflammation observed in IBD. Furthermore, the presence of activated CD8+ T cells in the gut mucosa has been linked to tissue damage and disease progression. These findings underscore the importance of assessing tissue-specific immune responses in IBD, as peripheral blood cell measurements may not fully capture the extent of mucosal immune dysregulation [5,6,7].

Our protocol integrates both techniques, providing a comprehensive immune profile of biopsy samples. This method allows for the identification, quantification, and spatial localization of lymphocytes and other immune cell subsets. In this study, we describe a workflow in which a single tissue sample is divided for both FC and pathological assessment, allowing direct comparison between methods. The goal of the current study is to demonstrate the feasibility of a paired, within-biopsy workflow enabling direct FC–IHC comparison with CD4/CD8 ratio. We also highlight the potential of our methodology for improving immune cell quantification in research and clinical applications.

## 2. Experimental Design

### 2.1. Materials

- Sterile single-pack CellTrics^®^ filters (Sysmex, Hyogo, Japan, Cat.no. 04-004-2323).
- Antibodies for immunohistochemistry:
  ○ CD3 (Clone SP7) (Zytomed Systems, Berlin, Germany);
  ○ CD4 (Clone SP35) (Cell Marque, Merck KGaA, Darmstadt, Germany);
  ○ CD8 (Clone C8/144B) (Dako, Agilent, Santa Clara, CA, USA).

- Monoclonal antibodies for flow cytometry (BD Biosciences, Franklin Lakes, NJ, USA):
  ○ CD45-APC (Clone 2D1);
  ○ CD3-PE (Clone SK7);
  ○ CD4-FITC (Clone SK3);
  ○ CD8-PerCP (Clone SK1).

### 2.2. Equipment

- BD FACSCaliBur Flow Cytometer (BD Biosciences).

## 3. Detailed Procedure

**Figure 1:**
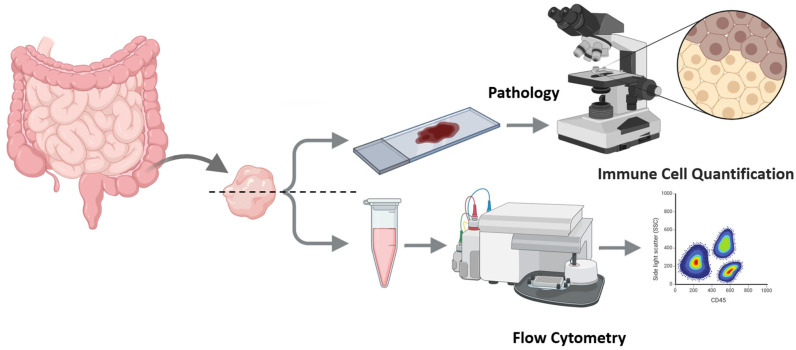
Workflow rationale for simultaneous assessment of lymphocytic subpopulations (such as CD4+ and CD8+ cells) with pathology and flow cytometry. Rationale high-level plan—Participants and sampling: individuals undergoing routine colonoscopy; mucosal pinch biopsies. Primary readout: CD4:CD8 ratio by IHC (cells/HPF) and by FC (% within CD3+ gate). Pairing: each biopsy split immediately into Part A (FFPE → H&E/IHC) and Part B (gentle mechanical dissociation → FC). QC thresholds: histology confirms mucosa-only; FC acquisition target ≥ 50,000 total events and ≥5000 CD45+ events. Statistics: Pearson correlation, Deming regression (error-in-variables), Bland–Altman with limits of agreement, and a simple heteroscedasticity check. Ethical approvals and consent: as stated below.

An overview of the protocol is outlined in Figure 1.

1. Tissue procurement, splitting, and parallel preparation (IHC and FC) from intestinal biopsies.

Specimen type and site: Obtain mucosal pinch biopsies (2–4 mm) during routine colonoscopy from predefined segments (terminal ileum, ascending, transverse, descending, sigmoid colon, and/or rectum; record exact segment and distance from anal verge). Samples with obvious submucosa/muscularis were excluded from analysis (confirmed on H&E). Following preparation, immediately upon excision, divide each sample into two portions, one for pathology assessment and one for flow cytometry (FC) analysis.

COMMENT: Split the specimen at the bench by a designated operator using a sterile scalpel on a chilled glass plate: part A (for pathology) and part B (for flow cytometry). Prevent mismatches with two pre-labeled tubes (A/B) with the same ID.

2. Pathology Assessment: Paraffin-embedded slides (prepared under standard conditions) were stained immunohistochemically with CD4 and CD8 antibodies.

Fixation and processing: Part A was immediately processed for FFPE following routine protocols.

Sectioning and staining: Serial 3–4 µm sections were cut; H&E was used to confirm mucosal representation and comparability with part B. IHC was performed for CD3, CD4, and CD8 using validated clones.

Quantification: Two blinded pathologists counted positive cells in non-overlapping HPFs (×400).

**Figure 2:**
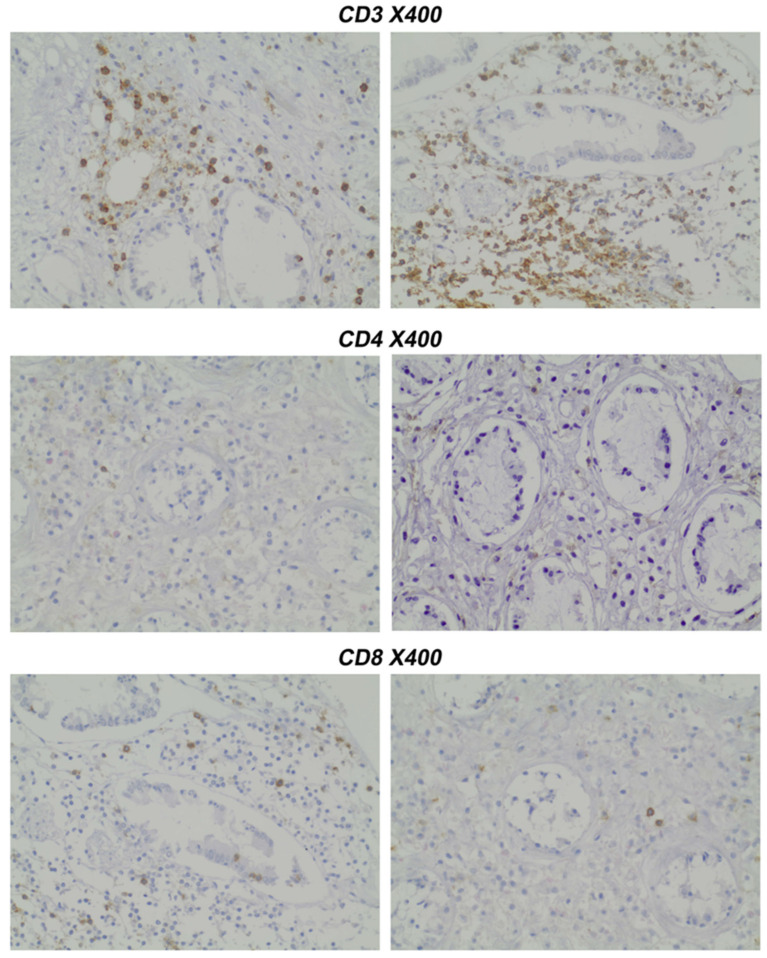
Representative figures from pathology assessment. Panels 1, 2, and 3 represent two independent immunohistochemical stains. For CD3, CD4, and CD8 cells, respectively (all in ×400 magnification). Counting was performed as positive cells per HPF by two blinded pathologists.

Results were assessed by two pathologists (EL and IT) independently, blinded for the patients’ clinical records and disease diagnosis, and scored according to the number of positive cells per high-power field. A representative staining is presented in Figure 2.

3. Sample preparation for Flow Cytometry (FC)
  3.1. Dissociate samples using a scalpel and 150 μL PBS buffer. Resuspend cells by gently pipetting 20–30 times to liberate infiltrating leukocytes; avoid harsh trituration/enzymes. Allow gravity to settle for 60 s; carefully transfer the supernatant to a fresh tube. Pass through a CellTrics^®^ filter (or analog) to remove tissue remnants and obtain a single-cell suspension.

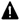
 CRITICAL STEP: The mix should be resuspended gently to avoid cell destruction. The main goal is to dissociate immune infiltrating cells (and to quantify them thereafter) from the tissue and not to prepare a single tissue cell suspension.

COMMENT: We intentionally do not perform a full enzymatic single-cell digestion. Instead, we use gentle mechanical dissociation to enrich dislodged infiltrating leukocytes, preserving surface epitopes and minimizing tissue-compartment bias. Cell dissociation protocols have been shown to affect cell type proportions [8,9].

  3.2. Aliquot 100 µL (or 1–5 × 10^5^ events where available). Incubate for 30 min at 4 °C in the dark with fluorochrome-conjugated antibodies: CD45-APC (leukocytes), CD3-PE (T cells), CD4-FITC, and CD8-PerCP.

COMMENT: Include single-stain controls for compensation and FMO/isotype controls as needed. Perform daily QC; apply compensation from single-stain controls.

  3.3. Lyse red blood cells by adding 1 × RBC lysis buffer, incubate for 2–3 min at RT, and analyze immediately white blood cell subpopulations. Acquire on FACSCalibur (or equivalent) with standard filters for FITC/PE/PerCP/APC. Targets: ≥50,000 total events and ≥5000 CD45^+^ events per sample whenever yield permits (record actuals).

COMMENT: An amount of 5000 analyzed cells are sufficient to obtain a clear quantitative view of blood cell subpopulations. Typically, 5000–10.000 cells can be obtained during the aforementioned procedure.

4. Flow Cytometry Acquisition and Analysis

Acquire data using a flow cytometer (in our case we used BD FACS Calibur) and analyze with appropriate software (such as CellQuest V3).

COMMENT: The selection of appropriate flow cytometry analysis software may be based on the available equipment. In our example, CellQuest V3.1 was used, which is the default software for a FACSCalibur Flow Cytometer. However, flow cytometry analysis software depends on the cytometer and the type of analysis.

  4.1. Gating strategy: CD45^+^ events were gated to identify leukocytes. CD3^+^ cells were gated as total T cells. CD4^+^ and CD8^+^ subsets were analyzed within the CD3^+^ population.

- CD45 vs. SSC-A→leukocyte gate.
- CD3 within CD45^+^→T-cell gate.
- CD4 vs. CD8 within CD3^+^→compute CD4:CD8 ratio; optionally report CD3^+^CD4^−^CD8^−^ fraction.
- Report % within parent and the CD4:CD8 ratio.

**Figure 3:**
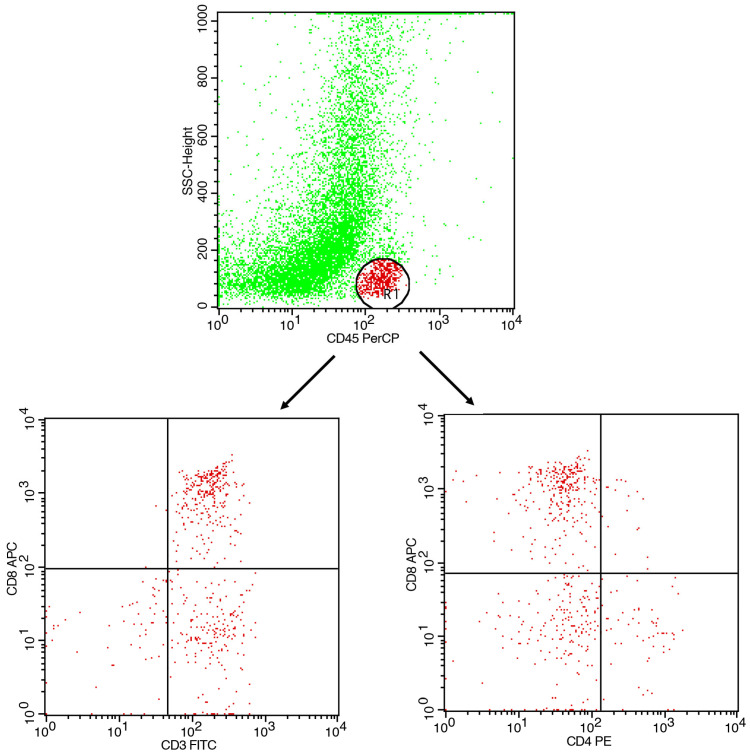
Flow cytometry analysis. A gating strategy for lymphocytes in the SSC/CD45 population is presented in the upper dot plots. Gated lymphocytes are then assessed for lymphocyte subpopulations. Characteristic populations (CD3/CD8 and CD4/CD8) are presented in the lower histograms.

A typical flow cytometry analysis of samples is presented in Figure 3.

COMMENT 1: Following the above gating strategy, FC reports proportions within CD3^+^ gates. Lymphocytes were identified using CD45 vs. SSC-A. Doublet discrimination can be applied (FSC area vs. FSC height) to ensure singlet purity. T cells can then be gated as CD3^+^ within CD45^+^, followed by CD4 and CD8 subsets.

COMMENT 2: In addition to the CD4^+^ and CD8^+^ fractions, the above analysis can identify the CD3^+^CD4^−^CD8^−^ double-negative subset. Although not further characterized here, this population may represent γδ T cells or innate lymphoid cells (ILCs), both of which are known to reside in colonic mucosa. Their presence highlights the potential of extended marker panels for more detailed mucosal immunophenotyping.

  4.2. Quality control: Include fluorescence-minus-one (FMO) controls and isotype controls.

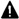
 CRITICAL STEP: Following standard quality control, use peripheral blood for standardization of leukocyte subpopulation in the instrument.

COMMENT: Keep the following troubleshooting list, referred to in the cytometry section:

- Low CD45^+^ events (<5000): increase gentle pipetting cycles; pool two rinse fractions.
- Excess RBCs: verify timely RBC lysis (2–3 min) and analyze immediately.

## 4. Expected Results

We include data from 10 individuals as a reference and proof-of-principle (Table 1). The samples taken were from the ascending colon. FC and IHC successfully quantified CD4^+^ and CD8^+^ T cells with clear gating strategies and tissue localization, respectively, complementing FC data. The relative percentage of CD4+ and CD8+ and the respective CD4:CD8 ratio have been calculated.

**Figure 4:**
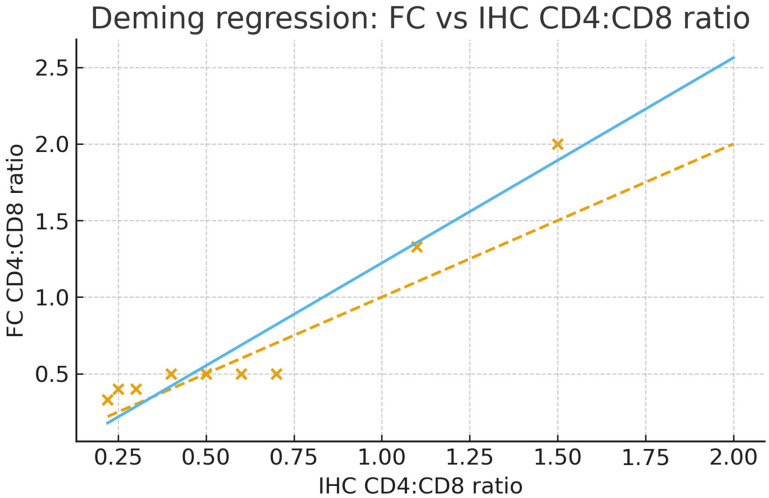
Deming regression comparing flow cytometry (FC) to immunohistochemistry (IHC) CD4:CD8 ratios (N = 10 paired biopsies). Pearson r = 0.956, *p* = 1.5 × 10^−5^. Deming fit (λ = 1): intercept = −0.117 (95% CI −0.330 to 0.330), slope = 1.340 (95% CI 0.292 to 1.520). Dashed line: identity (FC = IHC). Solid line: Deming fit.

**Figure 5:**
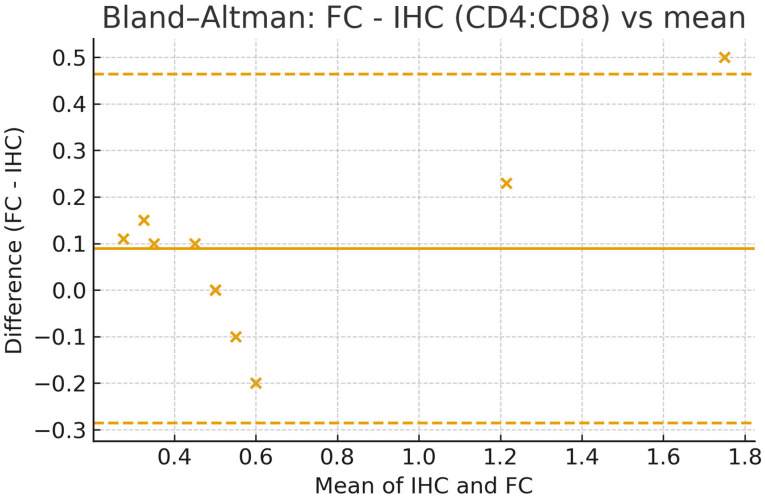
Bland–Altman analysis of agreement between FC and IHC CD4:CD8 ratios. Mean bias (FC−IHC) = 0.089; limits of agreement −0.285 to 0.463. A simple heteroscedasticity check (|difference| vs. mean) indicated non-constant variance (*p* = 0.0015).

We demonstrate the feasibility of a paired, correlative workflow that splits the same intestinal mucosal biopsy for parallel flow cytometry (FC) and immunohistochemistry (IHC), enabling direct method-to-method comparison on identical tissue. Across 10 paired samples, FC and IHC strongly correlated for the CD4:CD8 ratio (Pearson r = 0.956, *p* = 1.5 × 10^−5^). Agreement analyses further supported comparability: Deming regression (FC on IHC) yielded an intercept of −0.117 (95% CI −0.330 to 0.330) and a slope of 1.340 (95% CI 0.292 to 1.520), consistent with a slightly steeper scaling of FC across the observed range shown in Figure 4. Bland–Altman analysis showed a small positive bias (FC − IHC = +0.089) with limits of agreement from −0.285 to +0.463 ratio units (Figure 5). A simple heteroscedasticity check indicated that absolute differences increase with the mean (*p* = 0.0015), i.e., agreement is tighter at lower ratios and widens moderately at higher ratios. The statistically significant correlation coefficient between the two quantification methods also indicates the high similarity of the results calculated from the two methods (Table 1). Collectively, the available data show that the proposed protocol reliably quantifies immune cell populations within colorectal mucosal biopsies.

Our results showed a strong correlation between FC-based immune cell quantification and histopathological assessment. Flow cytometry successfully identifies CD4^+^ and CD8^+^ T cells with high specificity and quantification accuracy, as compared to pathology assessment. The BD FACS Calibur cytometer effectively detected and quantified CD45^+^ leukocytes, CD3^+^ T cells, and their subsets (CD4^+^ helper T cells and CD8^+^ cytotoxic T cells). FC provided rapid, quantitative immune cell analysis, making it particularly useful for assessing overall immune composition. Histopathological assessment provides spatial localization of CD4+ and CD8+ immune cells and confirms the presence of these immune cells within tissue architecture. The combined approach enabled direct cross-validation between FC and pathology in our sample data. FC provided an accurate quantitative assessment, while histology confirmed tissue distribution of the same cell types. To our knowledge, this is the first protocol of combined assessment of immune sub-population with FC and IHC. The diagnostic benefit is cross-validation from the same biopsy and a practical workflow when tissue is scarce.

Significantly, the CD4:CD8 ratio is a general marker of immune system balance and function, with a low ratio indicating immune activation or dysfunction, such as in aging-related immune decline (“inflammaging”) [10]. In addition to its role in blood, the CD4:CD8 ratio in tissues can provide deeper insight into local immune dynamics, particularly in lymphoid organs and mucosal sites. Studies suggest that tissue-based CD4:CD8 ratios may remain altered despite normalized ratios in blood, reflecting compartmentalized immune dysfunction [11,12]. A study by Geng et al. shows that in a population of 116 ulcerative colitis patients, those with a lower CD4/CD8 ratio (driven by an increase in activated CD8^+^ and CD3^+^HLA-DR^+^ T cells) tend to have more severe diseases. Importantly, this skewed ratio was associated with a higher risk of readmission or surgery, highlighting its potential prognostic value in clinical management [13]. The CD4/CD8 ratio also has implications as a biomarker in IBD, and their IHC density may also associate with the prognosis of colorectal cancer [5,14]. In addition, Lopez et al. show that colitis induced by immune checkpoint inhibitors is characterized by a predominant infiltration of CD8^+^ T cells, leading to a lower CD4/CD8 ratio compared with normal mucosa. The resulting immune profile and infiltration pattern closely resemble ulcerative colitis, underscoring that CD8-driven imbalance is central to checkpoint inhibitor colitis [15]. Τissue-resident memory T cells exhibit a spatiotemporally imprinted diversity [16] and may also act as potential targets for therapeutic interventions [17]. The speed of phenotyping is also critical for timely clinical decision-making [18,19].

These findings support the robustness and reproducibility of the flow cytometric component of the protocol, with IHC as the gold standard. Future implementation of paired histopathological quantification is expected to validate the immunophenotypic results, allowing for broader use of the method in translational and clinical research involving intestinal immune profiling. Advantages of the combined method include the fact that FC provides high-throughput quantification, while pathology ensures additional spatial resolution. Direct sample correlation improves the accuracy of immune cell profiling from both methodologies. In addition, FC can readily incorporate several additional markers for differentiation (such as CD45RA vs. CD45RO), activation (such as CD69, CD25, and HLA-DR), exhaustion (PD-1/CD279, TIM-3, and CD39), or tissue residency (CD49Aa and CD103), among others, in the same assay [20,21]—capabilities that are less practical with routine IHC. This multiplexing enables richer immunophenotyping from minimal biopsy material. The potential to identify a CD3^+^CD4^−^CD8^−^ cell fraction in our gating strategy is noteworthy, as it likely encompasses γδ T cells and/or ILCs, which play important roles in mucosal immunity [22]. While our panel was limited to CD3, CD4, and CD8, this observation underlines the feasibility and value of expanding future FC panels to capture these additional lineages. Novel panels of polyparametric and/or spectral flow cytometry may further add value to the diagnostic impact of our methodology, based on established methods [23,24].

Methodologically, the paired approach is complementary rather than substitutive: FC delivers rapid, multiparametric quantitation from minimal material, whereas IHC contributes spatial context and clinical familiarity, achieving a practical harmonization. As a proof-of-principle, our small pilot is not powered for definitive claims across the full CD4:CD8 spectrum; nevertheless, the combination of very high correlation, modest bias, and clinically interpretable limits of agreement supports the diagnostic utility of running FC alongside standard pathology on the same biopsy. We aim at future work that will (i) expand the cohort—particularly at higher CD4-dominant ratios and CD8-predominant tissues—to stabilize agreement across the full range, and (ii) leverage FC’s multiplex capacity (activation/exhaustion and tissue-resident markers) to enhance the biological and diagnostic granularity obtainable from limited intestinal biopsies.

While challenges such as cell loss during processing, differences in quantification metrics, and histological staining variability remain, our study demonstrates that this integrated method enhances immune profiling accuracy. We foresee that future refinements, including automated image analysis, AI-driven data integration, and validation across multiple disease models, might further improve the utility and applicability of this technique. Ultimately, this combined methodology offers a powerful tool for immunologists, pathologists, and clinicians, paving the way for improved immune diagnostics, biomarker discovery, and personalized therapeutic strategies.

## 5. Conclusions

Accurate immune cell quantification is essential for understanding the immune landscape in both health and disease. Our study presents a combined flow cytometry (FC) and pathology-based approach to characterize immune cell subpopulations from the same tissue sample, providing complementary advantages in immune profiling. This proof-of-principle study demonstrates the feasibility of integrating FC-based single-cell analysis with histopathology-based spatial resolution to yield a more comprehensive assessment of immune cell populations.

## Figures and Tables

**Table 1:** Correlation between CD4: CD8 ratio based on immunohistochemistry (IHC) and flow cytometry (FC) analysis. (** for a significant correlation with a *p* value < 0.001).

		**cd4:cd8 (FC)**
cd4:cd8 (FC)	Pearson Correlation	1
	Sig. (2-tailed)	
	Sum of Squares and Cross-products	1.486
	Covariance	0.165
	N	10
cd4:cd8 (IHC)	Pearson Correlation	0.956 **
	Sig. (2-tailed)	0.000
	Sum of Squares and Cross-products	1.881
	Covariance	0.209
	N	10

## Data Availability

The data obtained are freely available in the main body of the publication.
